# Identification of a Dual-Specific T Cell Epitope of the Hemagglutinin Antigen of an H5 Avian Influenza Virus in Chickens

**DOI:** 10.1371/journal.pone.0007772

**Published:** 2009-11-10

**Authors:** Hamid R. Haghighi, Leah R. Read, S. M. Mansour Haeryfar, Shahriar Behboudi, Shayan Sharif

**Affiliations:** 1 Department of Pathobiology, University of Guelph, Guelph, Ontario, Canada; 2 Department of Microbiology and Immunology, The University of Western Ontario, London, Ontario, Canada; 3 Institute of Hepatology, University College London, London, United Kingdom; New York University School of Medicine, United States of America

## Abstract

Avian influenza viruses (AIV) of the H5N1 subtype have caused morbidity and mortality in humans. Although some migratory birds constitute the natural reservoir for this virus, chickens may play a role in transmission of the virus to humans. Despite the importance of avian species in transmission of AIV H5N1 to humans, very little is known about host immune system interactions with this virus in these species. The objective of the present study was to identify putative T cell epitopes of the hemagglutinin (HA) antigen of an H5 AIV in chickens. Using an overlapping peptide library covering the HA protein, we identified a 15-mer peptide, H5_246–260,_ within the HA1 domain which induced activation of T cells in chickens immunized against the HA antigen of an H5 virus. Furthermore, H5_246–260_ epitope was found to be presented by both major histocompatibility complex (MHC) class I and II molecules, leading to activation of CD4+ and CD8+ T cell subsets, marked by proliferation and expression of interferon (IFN)-γ by both of these cell subsets as well as the expression of granzyme A by CD8+ T cells. This is the first report of a T cell epitope of AIV recognized by chicken T cells. Furthermore, this study extends the previous finding of the existence of dual-specific epitopes in other species to chickens. Taken together, these results elucidate some of the mechanisms of immune response to AIV in chickens and provide a platform for creation of rational vaccines against AIV in this species.

## Introduction

Avian influenza virus (AIV) is a type A influenza virus, which belongs to the family of enveloped RNA viruses. AIV genome encodes 11 proteins, among which hemagglutinin (HA) and neuraminidase (NA), are two surface antigens that are used to classify influenza viruses [1]. Birds are the natural hosts of AIV; however, infection in mammals, including humans can also occur by influenza viruses originating from avian hosts [2].

Immunity to influenza viruses is a concerted effort of both innate and adaptive responses.

In this regard, T cell-mediated immune responses play a critical role in defense against influenza infection [3]. A number of studies using mouse models have shown the induction of virus-specific CD8+ T cells following infection with influenza virus, and have underscored the important role of these cells in protection against influenza [4], [5]. CD4+ T cells also play a part in immunity against influenza. In fact, CD4+ T cells are induced following influenza virus infection and have a central role in immunity via the induction and maintenance of CD8+ T cell memory, and providing help to B cells for antibody production [6]–[8].

Numerous T cell epitopes from various proteins of influenza virus have been identified in human and mouse [9]. A number of studies have also been conducted to reveal the immunogenicity and protective effect of several of these epitopes. For example, epitopes derived from nucleoprotein (NP), polymerase acidic (PA) and M proteins of influenza virus induced strong specific cytotoxic T cell response [10]–[13].

Despite extensive research conducted on immune responses against influenza in mammals, our understanding of immunity, especially T cell responses, against influenza virus in chickens is very limited. It is known that AIV surface proteins including HA and NA are able to induce neutralizing antibodies in chickens and these antibodies play a role in protection against highly pathogenic avian influenza viruses (HPAI) [14]. The protective role of CD8+ T cells in AIV infection has also been shown [15]. It was demonstrated that depletion of CD8+ T cells in immunized birds resulted in abrogation of immunity against a challenge with a highly pathogenic H5N1 AIV [15]. Seo and Webster [16] have also shown that chickens immunized with the H9N2 subtype are protected against H5N1 AIV, indicating the effective recognition of the internal components of the virus by cells of the immune system. Interestingly, these birds mount a cross-reactive cytotoxic T lymphocyte (CTL) response and upon transfer of T cells from protected birds, naïve recipients become protected against challenge with virulent H5N1 virus [16]. The antigen specificity of these T cells is unknown. In fact, there is no information about MHC class I and class II-restricted epitopes of AIV recognized by chicken T cells. Furthermore, the effector responses of chicken T cells against AIV have not been fully elucidated, however, it has recently been shown that AIV infection induces up-regulation of cytokines such as IFN-γ [17].

The HA protein is known to be the most protective antigen of influenza virus [18]–[20] and has been used as a target antigen for a number of influenza vaccines in humans and animals [21]–[23]. Over 150 B cell epitopes as well as 113 CD4+ T cell and 35 CD8+ T cell epitopes have been identified within this antigen [9] and some of these epitopes have been shown to induce immune response and confer protection in humans [24], [25], mice [26], and rabbits [27]. However, there is no information available on epitopes of HA, or any other AIV proteins, that are recognized by chicken T cells. Therefore, in the present study, we sought to identify T cell epitopes of the HA protein of an H5 AIV in chickens. To our knowledge, this is the first report that describes a T cell epitope of AIV in avian species.

## Materials and Methods

### Chickens and Housing

Fertile SPF eggs were obtained from the genetically-defined line P2a (homozygous for the B^19^ haplotype) (Cornell University, Ithaca, NY, USA). This chicken line was used, because these chickens are homozygous for the B^19^ MHC haplotype and, moreover, we had developed reagents for this particular MHC haplotype. All birds were maintained in Horsfall units within an isolation unit. This research was approved by the University of Guelph Animal Care Committee and complied with the guidelines of the Canadian Council on Animal Care.

### Recombinant Vaccine

The TROVAC^™^-AIV H5 vaccine was kindly provided by Merial (Merial Select Inc, Gainesville, GA, USA). This vaccine is a recombinant fowlpox virus vector that expresses the HA antigen of the isolate A/turkey/Ireland/1378/83 (H5N8).

### Peptide Library

A library of 112 overlapping peptides, covering the entire HA protein encoded by the TROVAC™-AIV H5 vaccine virus, was synthesized by Mimotopes (Clayton, Australia). Peptides were 15 residues long and overlapped by 10 residues. Peptide pools were prepared at 10 peptides per pool for a total of 12 pools (pools 11 and 12 had 6 peptides per pool). For subsequent experiments, peptides were synthesized at a purity of 90% and suspended to 2.25 mM using the solvent recommended by the manufacturer. Purity of peptides was confirmed by mass spectrometry.

### Experimental Design

P2a chickens were vaccinated with TROVAC™-AIV H5 (or received vaccine diluent) at 14 days of age and received the secondary immunization on day 21 of age, at the dose recommended by the manufacturer. At 10 or 21 days post-secondary immunization, spleens were harvested and mononuclear cells prepared for *in vitro* stimulation with peptides. Following *in vitro* stimulation, cells were analyzed for indicators of cell activation either by assessment of tritiated thymidine incorporation or cytokine expression. MHC restriction of potential T-cell epitopes was assessed by blocking with anti-MHC or anti-CD4/anti-CD8 antibodies. Alternately, mononuclear cells were sorted into CD4+ and CD8+ subsets and cell proliferation was measured in the presence of irradiated antigen presenting cells and the cognate peptide. In both experiments, cell proliferation was measured by assessment of tritiated thymidine incorporation. Cytokine expression was also measured in CD4+ and CD8+ subsets sorted after exposure to the stimulatory peptide or the control treatment.

### Single Cell Suspension of Spleen and Separation of Mononuclear Cells

Spleen mononuclear cells were isolated as previously described [28]. Following isolation, cells were suspended in FACS buffer (1% bovine serum albumin in PBS) or complete RMPI cell culture medium.

### In Vitro Stimulation of Spleen Cells Using Peptide Pools and Individual Peptides

The initial screening of peptide pools was done on day 10 and day 21 post-secondary vaccination. In two replicate experiments, mononuclear cells were prepared from pools of three spleens from each of control and vaccinated birds. Cells were suspended in cell culture medium and peptide pools were added to a final concentration of 1.0 or 10 µM (per peptide). The peptides in pool 5 were screened individually at 1 µM and 10 µM in 3 replicates of pooled splenocytes from 3 vaccinated birds. The stimulatory peptide within pool 5 was titrated to determine a dose response, by addition of 0.1 µM through 20 µM of the peptide in replicate experiments using spleen cells from 4 individual vaccinated birds. Spleen cells were also treated with concanavalin A (ConA) (Sigma Aldrich, St. Louis, Mo) at 20 µg/ml (positive control), or with peptide solvent or medium as negative controls. Following addition of peptide or control treatments, cells were incubated at 41° C for 24, 48 or 72 hours. Eighteen hours before the end of each timepoint, 1 µCi of methyl-3H-thymidine, 25 Ci/mmol (GE Healthcare, Baie d'Urfe, Quebec) was added to each well. Plates were frozen at −80°C until processed. Cells were harvested onto glass fibre filters and thymidine incorporation was measured using the TopCount NXT Microplate Scintillation Counter (Packard/PerkinElmer, Waltham, Massachusetts, USA). Results are presented as stimulation index (SI) which is the mean cpm of treatment culture divided by the mean cpm of control cultures. SI values >3 for both concentrations of peptides were considered significant.

### Determination of MHC Restriction of T Cell Responses to the Putative Epitope

To determine MHC restriction of the response to the stimulatory peptide, spleen mononuclear cells from vaccinated birds (in 3 replicate experiments) were isolated and treated with the following antibodies: mouse anti-chicken MHC Class I and Class II, anti-CD4 and anti-CD8 antibodies or isotype control at a final concentration of 5 µg/ml for 90 minutes, before the addition of peptide (1 µM), or control treatments. Cells were incubated for 72 hours with an 18 hour pulse of methyl-3H-thymidine. All monoclonal antibodies were mouse anti-chicken and included anti-MHC II (EP25, IgG_1_, kindly provided by Dr. Michael Ratcliffe), anti-MHC I (clone F21-2, IgG_1_), anti-CD4, (clone CT-4, IgG_1_), anti-CD8α, (clone CT8, IgG_1_) and the isotype control (mouse IgG_1_) (all from Southern Biotech, Birmingham, Alabama).

### Fluorescent-Activated Cell Sorting (FACS) and Magnetic-Activated Cell Sorting (MACS)

For sorting, 6×10^7^ cells from vaccinated birds were double stained with mouse anti-chicken CD4-RPE and mouse anti-chicken CD8α-chain-FITC. FACS controls (1×10^6^ cells), included unstained cells and cells stained with either mouse anti-CD4-RPE, mouse anti-CD8α-chain-FITC or appropriate isotype controls. All antibodies were used at a concentration of 100 ng per 1×10^6^ cells in 100 µl. After staining, the labeled cells were immediately sorted using the FACSAria instrument (BD Biosciences, Franklin Lakes, NJ, USA) and used in the antigen presentation assay or used for RNA extraction.

In 3 replicate experiments, CD8+ and CD4+ spleen cells were magnetically sorted from vaccinated birds using the MACS system (Miltenyi Biotech, Auburn, California, USA). Spleen mononuclear cells (2×10^8^) were cultured for 24 hours with the peptide H5_246–260_, or the peptide solvent. Cells were split, washed in FACS buffer and labeled with mouse anti-chicken CD4 or mouse anti-chicken CD8α-chain (Southern Biotech, Birmingham, Alabama) in separate tubes, followed by a secondary staining with goat anti-mouse IgG microbeads (Miltenyi Biotech, Auburn, California, USA). RNA was extracted from sorted cell subsets.

### Preparation of Antigen Presenting Cells (APCs)

Mononuclear cells from the spleen of a vaccinated bird were prepared as the source of APCs. Cells were briefly pulsed with peptide before irradiation. Peptide (10 µM) or peptide solvent was added to 1×10^8^ cells and cell suspensions were incubated for 1 hour at 41° C in a humidified incubator. Cells were then irradiated with 30 Gy of gamma irradiation using a T780C Cobalt 60 radiation therapy treatment unit (Theratronics International, Kanata, Ontario).

### Antigen Presentation Assay

In two replicate experiments using three individual vaccinated birds, peptide-pulsed or non-pulsed APCs (1×10^6^ cells or 2×10^5^ cells in 50 µl) were mixed with CD4+ or CD8+ sorted splenocytes (1×10^5^ cells per 100 µl) to give an APC to responder ratio of either 10∶1 or 2∶1. Negative controls included sorted cells alone, with or without peptide (10 µM), or with ConA. Cell proliferation was measured at 72 hours.

### Gene Expression in Peptide-Stimulated Spleen Cells

Expression of interferon (IFN)- γ, interleukin (IL)-10, granzyme A and perforin mRNA was examined in spleen cells after stimulation with peptide. Spleen mononuclear cells of three control and five vaccinated birds were treated with peptide (10 µM), ConA or medium, and harvested at 2, 8, 16, and 24 hours post-stimulation for extraction of RNA. Total RNA from spleen mononuclear cells was extracted as described previously [29].

In a separate experiment, splenocytes from three vaccinated birds were stimulated for 24 hours with peptide (10 µM). Cells were stained with anti-CD4/anti-CD8 as described above and sorted into CD4+ and CD8+ subsets using MACS and RNA extracted using RNeasy® Micro extraction kit (Qiagen, Mississauga, Ontario).

A total of 1 µg of RNA was reverse transcribed to cDNA with oligo(dT)_12–18_ primers using the Superscript First-Strand cDNA Synthesis kit (Invitrogen Corporation, Carlsbad, CA, USA).

Primers, plasmid calibrator constructs and standard curves for real-time PCR were produced, as previously described [29], [30], [31], for β-actin, IL-10, IFN-γ, granzyme A, and perforin. To quantify the relative expression of cytokines, 10-fold diluted cDNA was used as the template for real-time PCR, using β-actin as the reference gene. The samples were amplified in 384-well plates using the Lightcycler® 480, or in glass capillaries using the Lightcycler® 2.0 (Roche Diagnostics GmbH, Mannheim, Germany) using reagents and conditions as previously described [29], [31].

### Data Analysis

Real-time PCR data was analyzed using REST-MCS© [32] to calculate the relative fold-change in gene expression between control and peptide stimulated samples. The data were normalized using the reference gene, β-actin, and are corrected for PCR efficiencies. PCR efficiencies were calculated previously [29], [30], [31]. The statistical significance level was set at p≤0.05.

### Sequence Analysis

Influenza virus sequences were obtained from GenBank and aligned with H5_246–260_ using NCBI BLAST. H5_246–260_ was compared to H5 sequences from strains of avian hosts (H5N1, H5N2, H5N3, H5N8 and H5N9) and avian/human hosts (H5N1). In addition, homology of H5_246–260_ with other hemagglutinin subtypes including strains of avian hosts (H1N1), avian/human hosts (H2N2), human hosts (H3N2), swine hosts (H3N2) and avian/human/swine hosts (H1N1) were examined.

The “Epitope Prediction” function of SYFPEITHI (http://www.syfpeithi.de/) was used to predict the HLA alleles that could bind to H5_246–260_. HLA alleles with a score of >15 are reported.

## Results

### Screening of the HA Peptide Library

**Figure 1:**
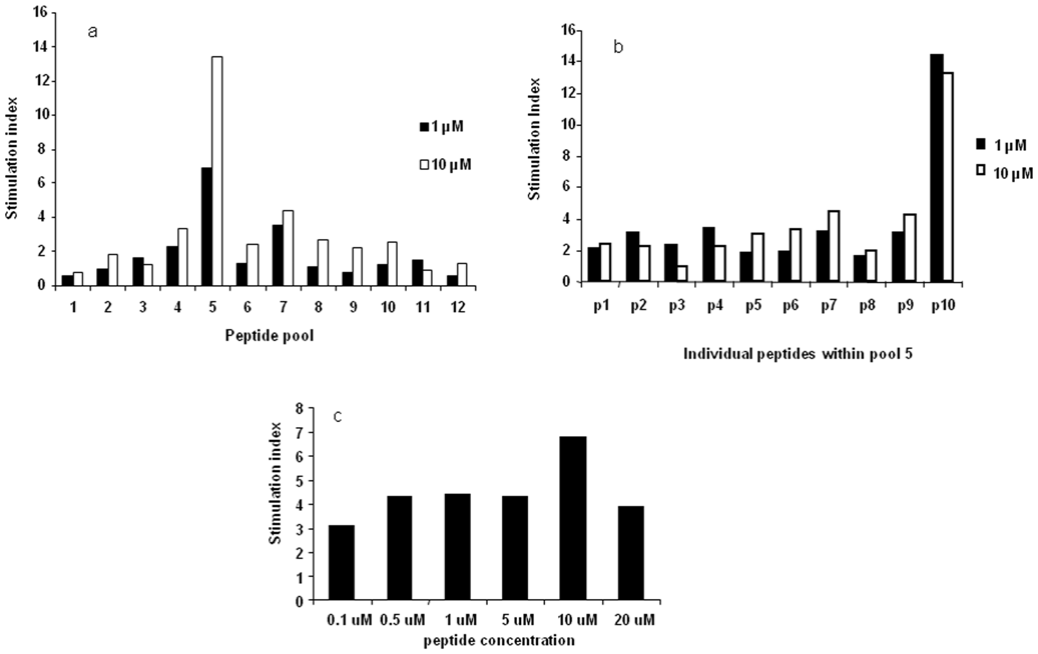
Identification of stimulatory peptide pools and individual peptides. P2a chickens were vaccinated with TROVAC^™^-AIV H5 or vaccine diluent. (1a) In two replicate experiments, at days 10 and 21 post-secondary vaccination, spleen mononuclear cells from control and vaccinated chickens were pooled and used for peptide screening. A peptide library covering the entire HA antigen of an H5 virus was used in 12 pools. Cells were stimulated with peptide pools at concentrations of 1.0 and 10 µM for 24, 48 or 72 hours. (1b) Peptides from pool 5 of the peptide library were used individually at day 21 post-secondary vaccination to stimulate spleen mononuclear cells of vaccinated P2a chickens. In each of three replicate experiments, cells from vaccinated birds were stimulated using peptide for 48 or 72 hours. (1c) Spleen mononuclear cells from vaccinated birds in 4 independent replicates were used to determine dose-dependent proliferative response to H5_246–260_. Cells were incubated with peptide or peptide solvent for 72 hours. For all figures, cell proliferation, as measured by average stimulation index, (mean cpm of treatment/mean cpm of peptide solvent cultures for all independent experimental replicates), is shown for spleen mononuclear cells harvested at 21 days post-secondary vaccination and cultured for 72 hours. SI values >3 for both concentrations of peptides were considered significant.

Spleen cells from chickens vaccinated with TROVAC™-AIV H5 consistently showed the highest proliferation in response to peptide pool 5 compared to other peptide pools and cells from sham immunized chickens. This response was observed at both 10 and 21 days post-secondary immunization and at peptide concentrations of 1.0 and 10 µM. (Figure 1a). To identify the cognate peptides, individual peptides within pool 5 were screened for activity. Peptide H5_246–260,_ which is located in the HA1 domain of the HA protein, induced greater cell proliferation compared to other peptides within the same pool or compared to controls (Figure 1b), suggesting that H5_246–260_ was the reacting peptide within this pool. The results were confirmed in independent experiments performed on different days. Titration of the stimulatory peptide revealed that spleen mononuclear cell stimulation occurred over a wide range of peptide concentrations, although in some of the experiments, using peptides at 10 µM induced a slightly higher level of proliferation in spleen cells (Figure 1c). Cell proliferation induced by H5_246–260_ was not a non-specific stimulation because cells from sham vaccinated birds did not proliferate in response to this peptide (data not shown). There was also a weak response to pool 7, but because it was not consistently present in both experimental replicates. Furthermore, the individual peptides within this pool did not display any stimulatory activities (data not shown). Therefore, this pool and its peptides were not examined any further.

### Cytokine Expression in Cells Stimulated with H5_246–260_ Peptide

**Figure 2:**
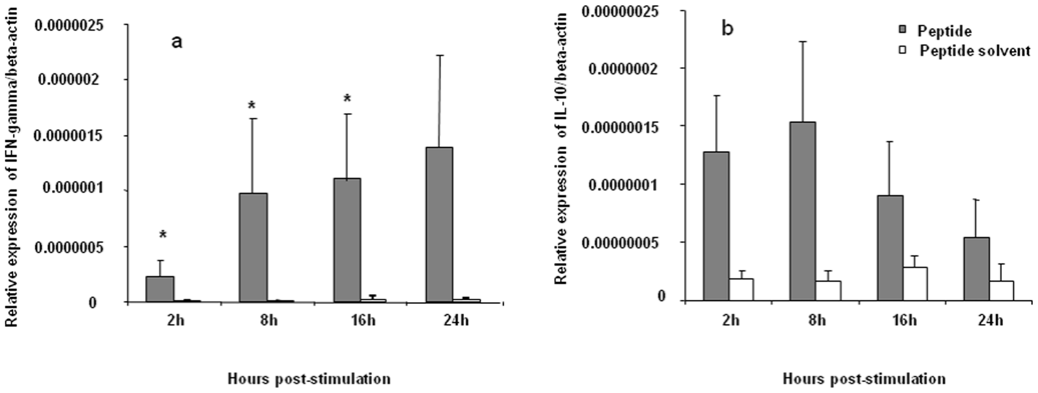
Cytokine mRNA expression in splenocytes in response to H5_246–260_. In three replicate experiments, spleen mononuclear cells from vaccinated birds were incubated with peptide or peptide solvent for 24 hours before RNA extraction and cytokine analysis using real-time PCR. Relative expression of IFN-γ (A), IL-10 (B) mRNA normalized to β-actin expression in stimulated and unstimulated spleen mononuclear cells are presented. Comparisons were made using the REST program and considered significant at P≤0.05 (*).

To further examine the recognition of H5_246–260_ peptide by T cells, the expression of IFN-γ and IL-10 genes was measured. IFN-γ and IL-10 were selected to represent the activation of CD4+ T helper (Th)1/CD8+ T cytotoxic (Tc)1 and regulatory T cells (Treg), respectively. IFN-γ expression was found to be higher in splenocytes stimulated with the identified peptide compared to untreated spleen cells. IFN-γ was significantly upregulated at 2, 8 and 16 hours post-stimulation compared to cells that were not treated with the peptide (p<0.001–0.05), whereas IL-10 expression was not significantly different between control and peptide stimulated cells. (Figure 2a,b). These findings suggest that Th1 or Tc1 cells, but not Treg, are activated by peptide stimulation.

### Determination of MHC Restriction Using Antibody Blocking

**Figure 3:**
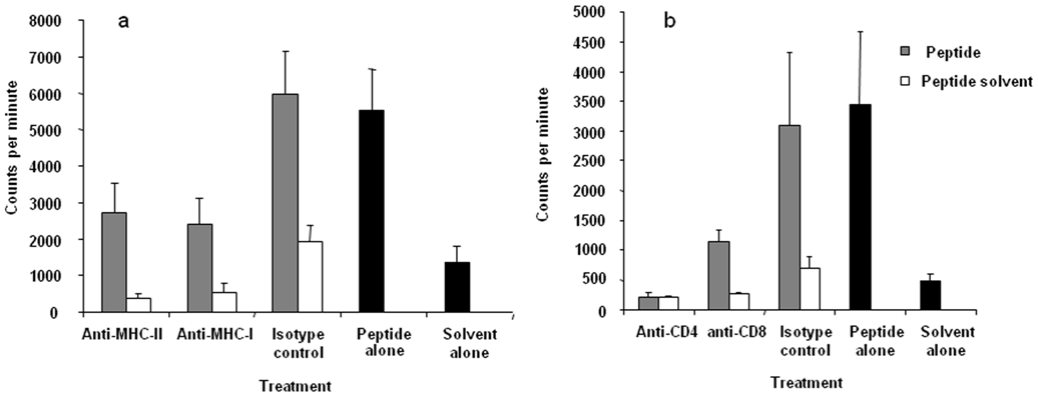
Determination of MHC restriction of response to H5_246–260_. Antibodies against MHC I and MHC II (A) and anti-CD4 and anti-CD8 (B) were used to block proliferative response to peptide, H5_246–260._ In at least 5 replicate experiments, spleen mononuclear cells from vaccinated birds were incubated with 5 µg/ml antibodies or isotype control before addition of peptide or solvent control. There were two additional groups of control cells, one being treated with the peptide without addition of any antibodies (peptide alone) and the second being treated with peptide solvent without addition of any antibodies (solvent alone). Proliferation was measured at 72 hours. Data are presented as counts per minute and error bars represent standard errors of the mean.

To examine MHC restriction of H5_246–260_ peptide, blocking experiments were conducted using antibodies against chicken CD4, CD8, MHC class I and class II. Using antibodies against MHC I and MHC II a reduction in cell proliferation in response to H5_246–260_ was noticed. Similar results were obtained in antibody blocking experiments using anti-CD4 and anti-CD8α antibodies (Figure 3a,b). Therefore, the peptide appeared to be presented to T cells via both MHC class I and II molecules.

### Activation of T Cell Subsets by H5_246–260_ Peptide-Pulsed APCs

**Figure 4:**
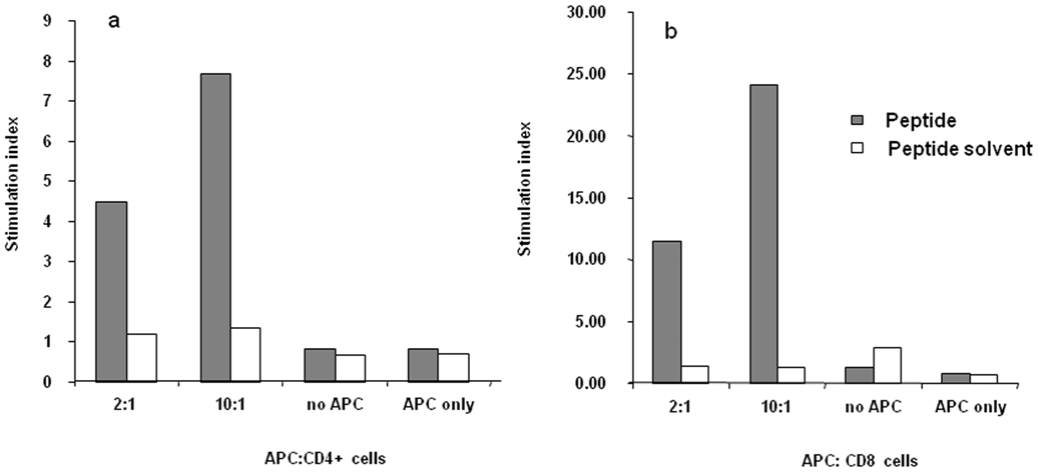
Proliferative response to H5_246–260_ in sorted CD4+ and CD8+ spleen cells. Spleen mononuclear cells from vaccinated birds, in 6 replicate experiments, were sorted into CD4+ and CD8+ T cell subsets followed by co-culture with APC and peptide, H5_246–260_ (10 µM) at an APC to responder ratio of either 2∶1 or 10∶1. T cell subsets were also cultured with or without the peptide in the absence APC (no APC). In another control group (APC alone), APCs were cultured in the absence or presence of the peptide. Proliferative response was measured at 72 hours. Results for CD4+ (A) and CD8+ (B) are presented as an average stimulation index, (mean cpm of treatment/mean of control for all independent experimental replicates).

To show that CD4 and CD8 T cell subsets can both recognize H5_246–260_ peptide presented by antigen presenting cells, CD4+ and CD8+ T cells were purified and stimulated *in vitro* with the cognate peptide. Stimulation of purified CD4+ or CD8+ T cells with the peptide-pulsed gamma-irradiated APCs resulted in a significant proliferation of both T cell subsets (Figure 4a,b). Also, when irradiated APCs alone were stimulated with the peptide in the absence T cells, no proliferation was observed (data not shown). These results confirm the stimulatory ability of H5_246–260_ peptide for both T cell subsets.

### Gene Expression in T Cell Subsets in Response to H5_246–260_ Peptide

**Figure 5:**
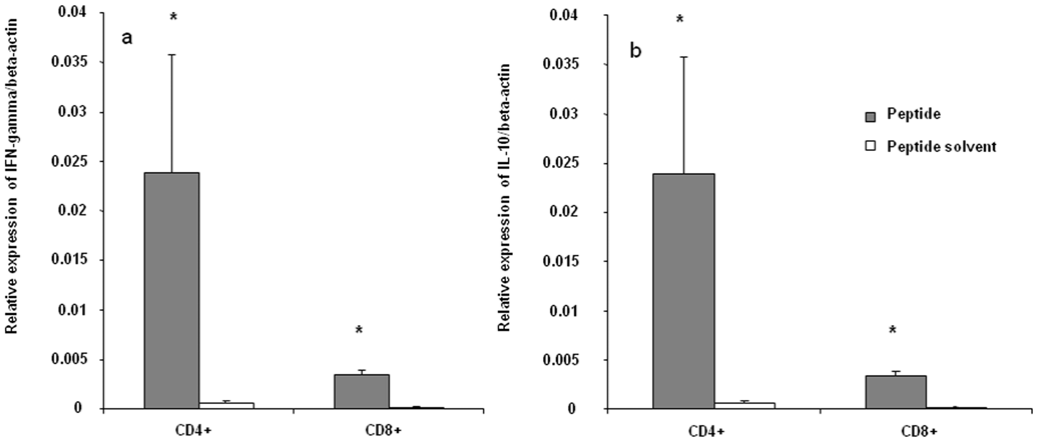
Cytokine mRNA expression in CD4+ and CD8+ T cell subsets. Spleen mononuclear cells from vaccinated birds in three replicate experiments, were co-cultured with peptide solvent or peptide, H5_246–260_ (10 µM) for 24 hours followed by magnetic cell sorting into CD4+ and CD8+ T cell subsets. Sorted cells were used for RNA extraction and cytokine analysis using real-time PCR of cDNA. Relative expression of IFN-γ (A), IL-10 (B) mRNA normalized to β-actin in CD4+ and CD8+ T cells treated with the peptide or peptide solvent are presented. Comparisons between peptide-treated and control cells were made using the REST program and considered significant at P≤0.05 (*).

**Figure 6:**
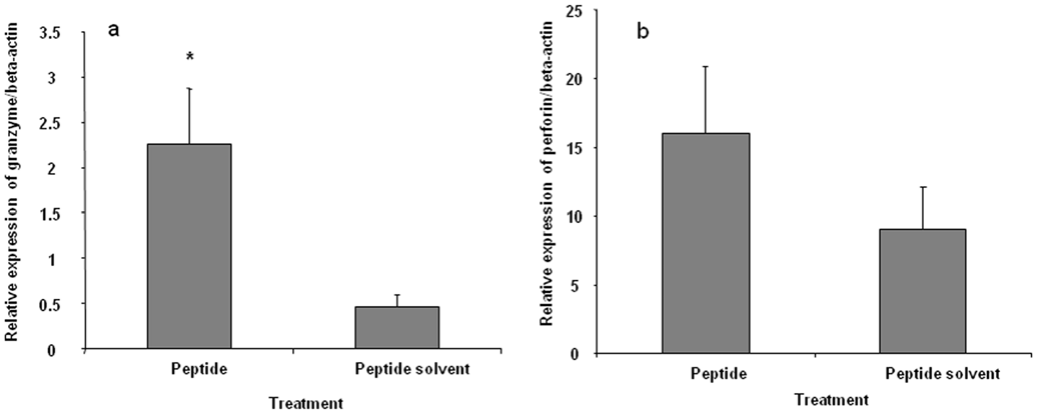
Granzyme A and perforin mRNA expression in CD8+ T cell subsets. Spleen cells from vaccinated birds in three replicate experiments, were co-cultured with peptide solvent or peptide, H5_246–260_ (10 µM) for 24 hours followed by magnetic cell sorting into CD4+ and CD8+ T cell subsets. Sorted cells were used for RNA extraction and cytokine analysis using real-time PCR. Relative expression of granzyme A (A), perforin (B) mRNA expression normalized to β-actin in CD4+ and CD8+ T cells treated with the peptide or peptide solvent are presented. Comparisons between peptide-treated and control cells were made using the REST program and considered significant at P≤0.05 (*).

T cell subsets were sorted after 24 hours of peptide stimulation and cytokine expression was determined in CD4+ or CD8+ T cells. Relative expression of both IFN-γ and IL-10 mRNA was significantly increased in both CD4+ and CD8+ subsets stimulated with H5_246–260_ peptide when compared to controls which received no peptide (p≤0.05) (Figure 5). There was also a significant (p≤0.05) increase in expression of granzyme A in CD8+ T cells stimulated with the peptide compared to the untreated cells. Expression of perforin was also slightly upregulated in the peptide-stimulated CD8+ T cells, although the difference between treated and control cells did not reach statistical significance (Figure 6).

### Sequence Analysis of H5_246–260_ Peptide

Peptide H5_246–260_ shared similarity with corresponding domains in other influenza viruses, including H5, H2N2, H3N2 and H1N1 viruses in the range of 46%-100% (Table 1). Based on peptide-binding motifs of the HLA alleles deposited in the SYFPEITHI database, H5_246–260_ is predicted to bind to HLA-A*01, A*0201, A*03, B*08, B*3902, and B*5101 as well as HLA-DRB1*0101, DRB1*0301, DRB1*0401, DRB1*0701, DRB*1501.

**Table 1:** Sequence Alignment of B7 peptide from HA of A/turkey/Ireland/1378/83 (H5N8) subtype with the corresponding peptides derived from HA of other influenza virus subtypes.

Peptide sequence	Virus strain	Subtype	Host	Similarity
WTILKPSDTINFESN	A/turkey/Ireland/1378/83	H5N8	Avian	100%
WTILKPSDTINFESN	A/duck/Ireland/113/1983	H5N8	Avian	100%
WTILKP**N**D**A**INFESN	A/turkey/England/50-92/91	H5N1	Avian	86%
WTILKP**N**D**A**INFESN	A/goose/Hong Kong/23/1978	H5N3	Avian	86%
WTILKP**K**D**A**INFESN	A/duck/Hong Kong/342/1978	H5N2	Avian	86%
WTILKP**N**D**A**INFESN	A/duck/Hokkaido/Vac-1/04	H5N1	Avian	86%
WTILKP**N**D**A**INFESN	A/Viet Nam/1194/2004	H5N1	Human, Avian	86%
WTILKP**N**D**A**INFESN	A/Hong Kong/156/97	H5N1	Human	86%
WT**L**L**DQG**DTI**T**FE**AT**	A/chicken/Hong Kong/14/1976	H1N1	Avian	53%
WT**L**L**E**PGDTI**I**FE**A**N	A/Texas/22/1990	H1N1	Human	66%
WT**L**L**DQG**DTI**T**FE**AT**	A/mallard/Alberta/35/1976	H1N1	Human, Avian, swine	53%
WT**LLDMW**DTINFES**T**	A/Japan/305/57	H2N2	Human, Avian	60%
WT**LLDMW**DTINFES**T**	A/Korea/426/1968	H2N2	Human	60%
WTI**V**KP**G**D**ILLIN**S**T**	A/Aichi/68/2007	H3N2	Human	46%
WTI**V**KP**G**D**ILLIN**S**T**	A/New York/232/2004	H3N2	Human	46%
WTI**V**KP**G**D**ILLIN**SN	A/Port Chlamers/73	H3N2	Human	53%
WTI**V**KP**G**D**VLVIN**SN	A/Human/Hong Kong/1/68	H3N2	Human	53%
WTI**V**KP**G**D**VLVIN**SN	A/England/42/72	H3N2	Human	53%

Bold-faced residues represent differences in the sequence of corresponding peptides compared to H5_246–260._

## Discussion

The main objective of the present study was to identify T cell epitopes of the HA antigen of an H5 AIV in chickens. Using a peptide library covering the full length of the HA protein, we found a 15-mer peptide that was able to induce T cell proliferation and IFN-gamma expression in chickens immunized with a fowlpox virus-based vaccine expressing the H5 protein. Our data suggest that this peptide was presented by both MHC class I and II molecules, leading to activation of CD4+ and CD8+ T cell subsets. The importance of these findings is two fold. First, this is the first study to identify an immunodominant T cell epitope of AIV in chickens and, second, the H5 epitope identified here has the capacity to stimulate both T cell subsets. Vaccination with dual-specific epitopes may be more efficacious than a mixture of CTL and Th cell epitopes, as they could allow CD4+ and CD8+ cells to interact with the same APC, thereby improving communication between cells [33].

In this study, we used a recombinant FPV-based vaccine expressing AIV-H5 from A/turkey/Ireland/1378/83 (H5N8). This vaccine has been shown to elicit antibody responses and protect chickens against a challenge with HPAI viruses [22], [18]. Here, we present evidence that this vaccine elicits T cell responses to the HA antigen in chickens.

Several T cell epitopes have already been identified for almost all proteins encoded by influenza A viruses in mouse and human. More specifically, close to 150 T cell epitopes, most of which are CD4+ T cell epitopes, have been identified within the HA antigen of different influenza virus subtypes [9]. In the present study, following screening 112 peptides covering the entire length of the HA protein, we identified a peptide epitope, H5_246–260,_ that elicited T cell proliferation and cytokine production. The H5_246–260_ peptide is located in the H1 region of HA and although its location does not contain receptor-binding sites of HA [34], it does constitute a glycosylation site.

The H5_246–260_ epitope is partially conserved (>86% similarity) among several H5 viruses. For example, A/Hong Kong/156/97 and A/Viet Nam/1194/2004 H5N1 viruses contain sequences within the same region of HA that are only different from the H5_246–260_ peptide by two residues at positions 7 and 9. Moreover, within the H5_246–260_ peptide, there are certain motifs that also appear to be conserved among other subtypes of influenza virus. For example, this epitope has sequence identity in the range of 53–66% with H1N1 viruses, 60% with H2N2 viruses and 46–53% with H3N1 viruses (9 and Table 1), despite the fact that HA is highly variable among influenza virus subtypes. A peptide epitope, IYWTIVKPGDILLINS, in this same region of HA of an H3N2 virus has been identified which is recognized by CD4+ T cells of individuals with HLA-DRB1*0701 or HLA-DRB1*0101 haplotypes [25], [9]. Although this remains a speculation, it is possible that the epitope identified in this study can elicit a T cell response against influenza viruses of other subtypes. Roti and colleagues [35] have already demonstrated that human subjects that were exposed to H1N1 and H3N2 viruses had cross-reactive CD4+ T cell responses to the HA antigen of A/Viet Nam/1203/2004 (H5N1).

Although there is no empirical evidence available, based on our analysis of anchor residues of H5_246–260_ and peptide-binding motifs of various HLA class I and class II alleles, H5_246–260_ may be able to bind several HLA alleles. These observations raise the possibility of using H5_246–260_ as a potential epitope for inclusion in future vaccines against H5N1 in chickens, and possibly for humans. However, this needs to be confirmed experimentally.

Despite the fact that the peptide library used in the present study covered the entire HA sequence, only one peptide pool was found to induce proliferation of cells in a consistent manner. Also, within this pool, only one peptide was shown to be stimulatory for T cells of vaccinated birds, suggesting that the identified peptide is an immunodominant epitope. This observation may indicate the narrow scope of chicken T cell responses to the HA antigen. An alternative, but not mutually exclusive scenario is that this may be a line-specific observation. Bennink and Yewdell [36] have reported that in mice carrying the H-2^k^ or H-2^d^ MHC haplotypes, CTLs can recognize HA only when it is presented in the context of K^k^ and K^d^, but not when presented by MHC class I D and L molecules. It is important to note that chickens have two MHC-I and MHC-II genes, the so-called major and minor genes, among which only the major genes are predominantly expressed [37]. This perhaps imposes some limitations on the repertoire of peptides recognized by chicken T cells.

The H5_246–260_ epitope was able to induce both CD4+ and CD8+ T cell responses. This induction was specific because the peptide had no stimulatory effect on cells of sham vaccinated birds and, moreover, the response was completely or partially blocked by antibodies against MHC-I, MHC-II, CD4, and CD8. In addition, both purified CD4+ and CD8+ cells responded to the peptide when it was presented to them by APCs, although APCs alone were not stimulated with the peptide. Presence of epitopes of infectious agents and cancer antigens with dual specificity for both CD4+ and CD8+ T cell subsets has previously been reported by us and others (38–43). In the context of cancer, dual specificity T cell epitopes have been identified for alpha-fetoprotein, NY-ESO-1, and carcinoembryonic antigens [38], [39], [40], [41]. Shams and colleagues (2004) discovered a 15-mer peptide (CFP10_71–85_) of the culture filtrate protein antigen of *Mycobacterium tuberculosis* that could induce production of IFN-γ by CD4+ T cells and CTL activity by CD8+ T cells. Furthermore, this peptide exhibited promiscuity in that it interacted with T cells of individuals with several different MHC genotypes [33]. A similar phenomenon has been observed for some of the epitopes of lymphocytic choriomeningitis virus, malaria, and HIV [40], [42], [43]. Dual specific epitopes have also been identified in influenza virus antigens. For instance, HA_252–271_ of influenza virus A/JAPAN/305/57 (H2N2), which is recognized by CTLs restricted to mouse H-2K^k^ is also recognized by CD4+ T cells restricted to I-A^d^ [44]. Interestingly, this peptide overlaps with the peptide (H5_246–260_) that we have identified in the present study. The observation that H5_246–260_ can activate both T cell subsets has some implications for the use of this epitope as a candidate vaccine against AIV in chickens.

Taken together, we have identified, for the first time, a dual specific epitope within the HA antigen of an H5 avian influenza virus, which is recognized by both CD4+ and CD8+ T cell subsets of chickens vaccinated against H5 protein. Our findings raise the possibility of using this epitope as a potential vaccine against H5 AIV in chickens. In fact, studies are now underway to assess the protective efficacy of the H5_246–260_ epitope in B19 as well as in non-B19 chickens.
